# Adaptive Responses of Common and Hybrid Bermudagrasses to Shade Stress Associated With Changes in Morphology, Photosynthesis, and Secondary Metabolites

**DOI:** 10.3389/fpls.2022.817105

**Published:** 2022-03-03

**Authors:** Yiqin Cao, Kang Yang, Wei Liu, Guangyan Feng, Yan Peng, Zhou Li

**Affiliations:** College of Grassland Science and Technology, Sichuan Agricultural University, Chengdu, China

**Keywords:** polyploidization, morphology, chlorophyll, photochemical efficiency, flavonoids, proanthocyanidin

## Abstract

Alteration of ploidy in one particular plant species often influences their environmental adaptation. Warm-season bermudagrass is widely used as forage, turfgrass, and ground-cover plant for ecological remediation, but exhibits low shade tolerance. Adaptive responses to shade stress between triploid hybrid bermudagrass cultivars [“Tifdwarf” (TD), “Tifsport” (TS), and “Tifway” (TW)] and tetraploid common bermudagrass cultivar “Chuanxi” (CX) were studied based on changes in phenotype, photosynthesis, and secondary metabolites in leaves and stems. Shade stress (250 luminance, 30 days) significantly decreased stem diameter and stem internode length, but did not affect the leaf width of four cultivars. Leaf length of CX, TD, or TW showed no change in response to shade stress, whereas shade stress significantly elongated the leaf length of TS. The CX and the TS exhibited significantly higher total chlorophyll (Chl), Chl a, carotenoid contents, photosynthetic parameters [PSII photochemical efficiency (Fv/Fm), transpiration rate, and stomatal conductance] in leaves than the TW and the TD under shade stress. The CX also showed a significantly higher performance index on absorption basis (PIABS) in leaf and net photosynthetic rate (Pn) in leaf and stem than the other three cultivars under shade stress. In addition, the TS maintained higher proantho cyanidims content than the TW and the TD after 30 days of shade stress. Current results showed that tetraploid CX exhibited significantly higher shade tolerance than triploid TD, TS, and TW mainly by maintaining higher effective photosynthetic leaf area, photosynthetic performance of PSI and PSII (Pn and Fv/Fm), and photosynthetic pigments as well as lower Chl a/b ratio for absorption, transformation, and efficient use of light energy under shade stress. For differential responses to shade stress among three triploid cultivars, an increase in leaf length and maintenance of higher Fv/Fm, gas exchange, water use efficiency, carotenoid, and proanthocyanidin contents in leaves could be better morphological and physiological adaptations of TS to shade than other hybrid cultivars (TD and TW).

## Introduction

Light is an indispensable ecological factor for plant growth and development. Weak or intense light affects plant morphogenesis and photosynthesis depending on different species, such as C_3_ and C_4_ plants. As compared to C_3_ plants, the C_4_ plants need more lights in their life cycle. Photosynthesis provides energy for plant life activities and also controls plant morphogenesis. However, net photosynthetic rate (Pn) always decreases with increasing shade, especially in C_4_ plants (Kubásek et al., 2013; Xie et al., 2020). The shade also affects other photosynthetic characteristics, such as inconsistent light saturation and compensation points, as well as declines in non-photochemical burst capacity and electron transfer rate (Huang et al., 2011; Kim et al., 2011). In addition to physiological changes including declines in photosynthesis, transpiration rate (Tr), and carbohydrate accumulation, shade induces morphological alterations, as demonstrated by thinner leaves and stems, longer leaves and internodes, lower shoot density, and more upright growth (Fankhauser and Batschauer, 2016). In nature, shade also modifies microenvironmental conditions leading to high air relative humidity, less air movement, and high soil moisture content, which promotes the possibility of disease occurrence. For most fruit trees and vegetables, the shade limits their yield and quality (Allan and Carlson, 2003; Stier, 2001). Horticultural plants including various flowers, ornamental grasses, and turfgrasses often suffer from more shade situations than trees since they have a lower ecological niche (Pires et al., 2011; Fan et al., 2019). Shade stress caused by tress or buildings decreases turf quality and also increases turf maintenance costs. Insufficient light affects crop growth and development resulting in a significant reduction in crop dry matter accumulation and yield (Demotes-Mainard and Jeuffroy, 2004), such as 20% reduction in rice yield, under low light (Estrada-Campuzano et al., 2008). Thus, the breeding and application of shade-enduring plants are of great significance to agriculture and horticulture.

Reactive oxygen species (ROS) caused by shade oxidize plant cell membranes leading to an increase in cell membrane permeability. To cope with weak light environments, plants have evolved multiple adaptive pathways involved in the regulation of plant growth, physiology, and metabolic changes (Xie et al., 2017; Esmailpourmoghadam and Salehi, 2021). An enhanced antioxidant defense system is one of the most effective pathways to remove ROS, thereby reducing oxidative damage under shade stress. Previous studies have reported that higher activities of antioxidant enzymes, such as superoxide dismutase (SOD), peroxidase (POD), ascorbate peroxidase (APX), and catalase (CAT), were associated with better shade tolerance in buffalograss (*Buchloe dactyloides*) (Ren et al., 2017) and centipedegrass (*Eremochloa ophiuroides*) (Zhou and Cao, 2006). Except for antioxidant enzymes, some of the organic metabolites including carotenoids (CR), flavonoids (FA), and proantho cyanidins (PC) with stronger antioxidant properties play vital roles in scavenging ROS to alleviate oxidative damage to cells in plants. CR has the ability to absorb and dissipate excess light energy, which is an important mechanism of photoprotection in plants (Takahashi and Badger, 2011; Son et al., 2021). In addition, the CR is also an excellent scavenging agent of single-linear oxygen (Zakar et al., 2016). It has been shown that the FA exhibits a stronger scavenging capacity for ROS than vitamin C and E analogs under stress conditions (Agati and Tattini, 2010). Plants that grow under low light increased CR and chlorophyll (Chl) b contents as compared to those plants that grow under normal conditions (Huang et al., 2011). The PC is a key component of phytochrome and is also known as a condensed tannin involved in defensive adaptation against biotic and abiotic stresses (Debeaujon et al., 2003). Plants accumulated more PC in the upper sunny leaves than that in the lower shady leaves (Mole et al., 1988). In addition, it has been shown that enhanced UV radiation-induced PC accumulation in birch (*Betula platyphylla*) seedlings (Anu, 1998).

During the process of plant evolution, polyploidization alters botanical morphology, which is characterized by taller shoots, bigger leaves, and improved biomass (Abdolinejad et al., 2021; Hu et al., 2021). Polyploidization also could be beneficial for plants to develop better adaption to various environmental stresses. For example, tetraploid sea barley (*Hordeum marinum*) materials exhibited better drought tolerance than their diploid ancestors (Zhou et al., 2019). Bermudagrass (*Cynodon dactylon*) is one of the most important and popular warm-season C_4_ grass species for landscape greening and sports turf (Li et al., 2011). Common bermudagrass is rich in wild germplasms with a coarse texture and aggressive rhizomes. Triploid hybrid bermudagrass has been developed by crossbreeding tetraploid common bermudagrass with diploid African bermudagrass (*Cynodon transvaalensis*), which produces many genotypes, such as “Tifdwarf,” “Tifsport,” and “Tifway,” with a finer texture and lower mowing height than common bermudagrass. These hybrid cultivars are more suitable for use in golf course and other athletic fields. However, one of the main disadvantages of hybrid bermudagrass cultivars is a weak tolerance to shade stress (Huang et al., 2019). It has been reported that approximately one-quarter of ground cover plants in the city suffer from shade stress, which is derived from tall buildings, broad-leaved trees, conifers, etc. (Bell et al., 2000). Richardson et al. (2019) concluded that the shade degraded the turf quality of bermudagrass and also increased the cost of management and maintenance of bermudagrass turf in football court. The study of Trappe et al. (2011) found that there were significant variations among hybrid bermudagrasses because “Princess 77” and “Riviera” showed higher coverage than “Patriot,” “Tifsport,” and “Tifway” when these cultivars were grown under some shade condition. However, differential responses to shade between leaf and stem, as well as a potential mechanism of shade tolerance between tetraploid and triploid bermudagrasses, are worth further investigation.

After more than 10 years of selective breeding, a new common bermudagrass cultivar “Chuanxi” (CX) was developed. The new cultivar exhibits a finer texture, lower mowing height, and longer green period than other common bermudagrass and could be used for landscaping and ecological remediation. Most of the previous studies mainly focused on leaves in response to shade, but the adaptive mechanism of stems was not fully understood under low-light conditions. The purpose of this study is (1) to evaluate shade tolerance of one tetraploid common cultivar CX and three triploid hybrid cultivars “Tifdwarf” (TD), “Tifsport” (TS), and “Tifway” (TF) and (2) to further reveal the mechanism of shade tolerance between common and hybrid bermudagrasses associated with phenotypic alteration, photosynthetic performance, and secondary metabolites. Current findings will provide effective information about the differential mechanism of shade tolerance between tetraploid and triploid plant species.

## Materials and Methods

### Plant Materials and Treatment

Tetraploid common bermudagrass (*Cynodon dactylon*) cultivar “Chuanxi” (CX) and three triploid hybrid bermudagrass (*Cynodon transvaalensis* × *Cynodon dactylon*) cultivars “Tifdwarf” (TD), “Tifsport” (TS), and “Tifway” (TW) were selected as experimental materials. Stoloniform stems were obtained from the resource nursery in the Research Farm of Sichuan Agricultural University (Chongzhou, Sichuan, China). Stoloniform stems were planted in white PVC tubes (33 cm high and 11 cm diameter) containing sands and soils (1:1, v:v). Each tube included six independent stems and each stem had three internodes. All the materials were cultivated and managed regularly including sand covering, pruning, watering, and fertilizing once a week in the greenhouse (average 29/25°C (day/night), 60% relative humidity, and 700–800 μmol⋅m^–2^⋅s ^–1^ PAR). To form a dense lawn, the mowing height of all materials was maintained at 1.5 cm. After two and a half months of establishment in the greenhouse, all materials were moved into growth chambers for 7 days of acclimation to growth chamber condition before stress treatment [30/26°C (day/night), 60% relative humidity, and 750 μmol⋅m^–2^⋅s ^–1^ PAR]. Then, half of the materials of each cultivar were transferred to low-light growth chambers for shade stress [30/26°C (day/night), 60% relative humidity, and 250 μmol⋅m^–2^⋅s ^–1^ PAR]. After 30 days of cultivation under normal conditions or shade stress, leaf and stem samples were taken for determination and data analysis. Each treatment included four independent biological replications (four tubes).

### Determination of Plant Growth and Water Status

Leaf length (LL) and leaf width (LW) of the first fully expanded leaf and stem internode length (SIL) of the third stem node were measured by using a ruler (accuracy of 1 mm); the stem diameter (SD) was measured by using a vernier caliper (accuracy of 0.02 mm). Stoloniferous stems were selected randomly from each pot for determination. The relative water content in the leaves was calculated by the formula RWC (%) = [(FW-DW)/(TW-DW)] × 100, where FW is the fresh weight, TW is the saturated fresh weight, and DW is the dry weight (Barrs and Weatherley, 1962). For the determination of osmotic potential (OP), collected fresh leaves and stems were immediately soaked in distilled water for 8 h. the water-saturated leaves and stems were immediately frozen in liquid nitrogen for 8 min and then completely thawed at 4°C. The OP of saps in leaves was measured by an osmotic potential instrument (Wescor, Logan, UT, United States). The OP was calculated based on OP (MPa) = −c × 2.58 × 10^–3^.

### Determination of Chlorophyll Content and Photosynthetic Parameters

For Chl content, 0.1 g fresh leaves or stems were soaked in a 10 ml extraction solution (95% ethanol: 80% acetone, volume 1:1) in the dark until all tissues turned white. The absorbance value of the extraction solution was determined at 470, 645, and 663 nm with a spectrophotometer (Spectronic Instruments, Rochester, NY) (Arnon, 1949). For the determination of Chl fluorescence parameters including photochemical efficiency of PSII (Fv/Fm) and performance index on absorption basis (PIABS), leaves were placed in the dark for 30 min by using the leaf clamping. Fv/Fm and PIABS were recorded by a Chl fluorescence system (Pocket PEA, Hansatech, Norfolk, United Kingdom). Photosynthetic parameters including net photosynthetic rate (Pn), Tr, stomatal conductance (Gs), and water use efficiency (WUE) were measured by a portable photosynthesis system (CIRAS-3, PP Systems, Norfolk, United Kingdom). Leaves or stems were placed in the leaf chamber (400 μl CO_2_ L ^–1^ and 800 μmol photon m ^–2^ red and blue light) for determination. Leaf temperature or relative humidity was maintained at 25°C or 60%, respectively. Leaf samples were then cut from plants and scanned with Magic W and TM Portable Scanner (PDS-ST415-VPS, VuPoint Solutions) to measure a leaf or stem area which was used to calculate Pn, Tr, Gs, and WUE (Liang et al., 2021).

### Determination of Organic Metabolites

Water-soluble carbohydrate (WSC) content was measured according to the method of Li et al. (2015). Briefly, dry leaves or stems were ground into fine powders and 0.05 g of the powders were extracted with 2 ml of 80% (V/V) methanol in 80°C water bath for 40 min. The extract was centrifuged at 5,000 *g* for 10 min, and the supernatant was taken for determining the WSC. The reaction mixture (1 ml of supernatant, 4 ml of 98% sulfuric acid, and 1 ml of 5% phenol) was heated to 100°C in a water bath for 10 min. The cooled reaction mixture was determined at 490 nm with D-glucose as the standard. Flavonids (FA) content (Art. No. G00118W) and proantho cyanidins (PC) content (Art. No. G0120W) were determined by using test kits (Suzhou Comin Biotechnology Company) according to the manufacturer’s instructions.

### Data Analysis

SPSS 25 (IBM, Armonk, NY, United States) was used for all data and PCA analyses. Significant differences among TD, TS, TW, and CX varieties were tested at *p* ≤ 0.05. Stress index (SI) was used to evaluate stress tolerance (SI = stress parameter/normal parameter × 100) (Zhang et al., 2020), which presented a relative change of one particular parameter between stress condition and normal conditions. Heat map data were processed as log_2_ (SI/100).

## Results

### Changes in Phenotype and Growth Parameters in Response to Shade

**FIGURE 1:**
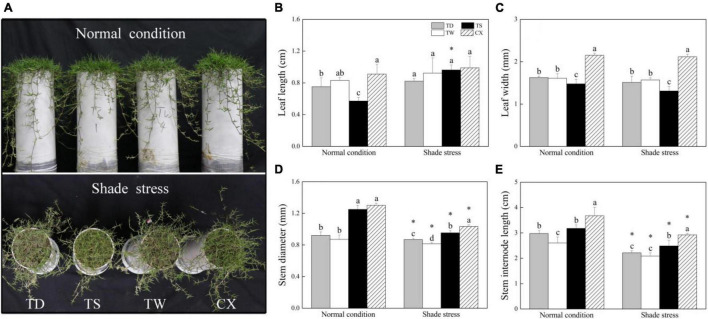
Effects of shade stress on **(A)** phenotype, **(B)** leaf length, **(C)** leaf width, **(D)** stem diameter, and **(E)** stem internode length of four bermudagrass cultivars. Vertical bars indicate the ± SE of mean (*n* = 5). Different letters above columns indicate significant differences (*p* ≤ 0.05) among the four cultivars under normal conditions or shade stress. The asterisk (*) above the letter indicates a significant difference (*p* ≤ 0.05) in one particular cultivar between normal conditions and shade stress.

Phenotypic changes showed that the leaf color of four different cultivars turned yellow after 30 days of shade stress (Figure 1A). Under normal conditions, TS had significantly the smallest LL and LW than the other three cultivars (Figures 1B,C). However, the LW and SIL of CX were significantly higher than the other cultivars under normal conditions (Figures 1C,E). There was no significant difference in LL among TD, TW, TS, and CX under shade stress (Figure 1B). Under shade stress, the LW, SD, and SIL of CX were significantly higher than those of the other three cultivars (Figures 1C–E). The LW of TS and the SD of TW were lowest than those of other cultivars under shade stress (Figures 1C,D). The decrement of SD and SIL was significant in all cultivars in response to shade stress (Figures 1D,E).

### Changes in Leaf Relative Water Content and Osmotic Potential in Response to Shade

**FIGURE 2:**
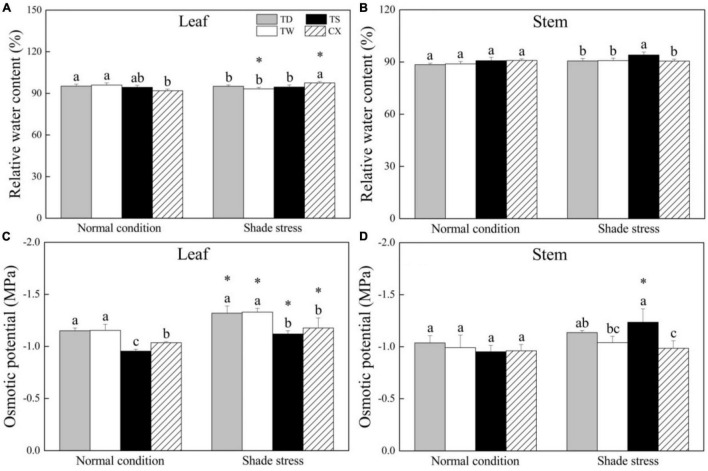
Effects of shade stress on **(A)** relative water content in leaf, **(B)** relative water content in stem, **(C)** osmotic potential in leaf, and **(D)** osmotic potential in the stem of four bermudagrass cultivars. Vertical bars indicate the ± SE of mean (*n* = 4). Different letters above columns indicate significant differences (*p* ≤ 0.05) among the four cultivars under normal conditions or shade stress. The asterisk (*) above letter indicates a significant difference (*p* ≤ 0.05) in one particular cultivar between normal conditions and shade stress.

Under normal conditions, the RWC in leaves of TD and TW was significantly higher than that in the leaves of CX, but CX exhibited significantly higher leaf RWC than the other three cultivars under shade stress (Figure 2A). The RWC in stems was not significantly different among four cultivars under normal conditions, and the TS showed significantly higher RWC than other cultivars in stems under shade stress (Figure 2B). There was no significant difference in OP between TW and TD under normal conditions and shade stress, and the OP in leaves of TW and TD was significantly lower than that in leaves of TS and CX under normal and shade stress conditions (Figure 2C). The shade stress significantly decreased OP in the leaves of all cultivars (Figure 2C). In stems, there were no significant differences in OP among the four cultivars under normal conditions (Figure 2D). Under shade stress, the TS maintained the lowest OP than the other three cultivars in stems (Figure 2D).

### Changes in Chlorophyll and Carotenoid Content in Response to Shade

**FIGURE 3:**
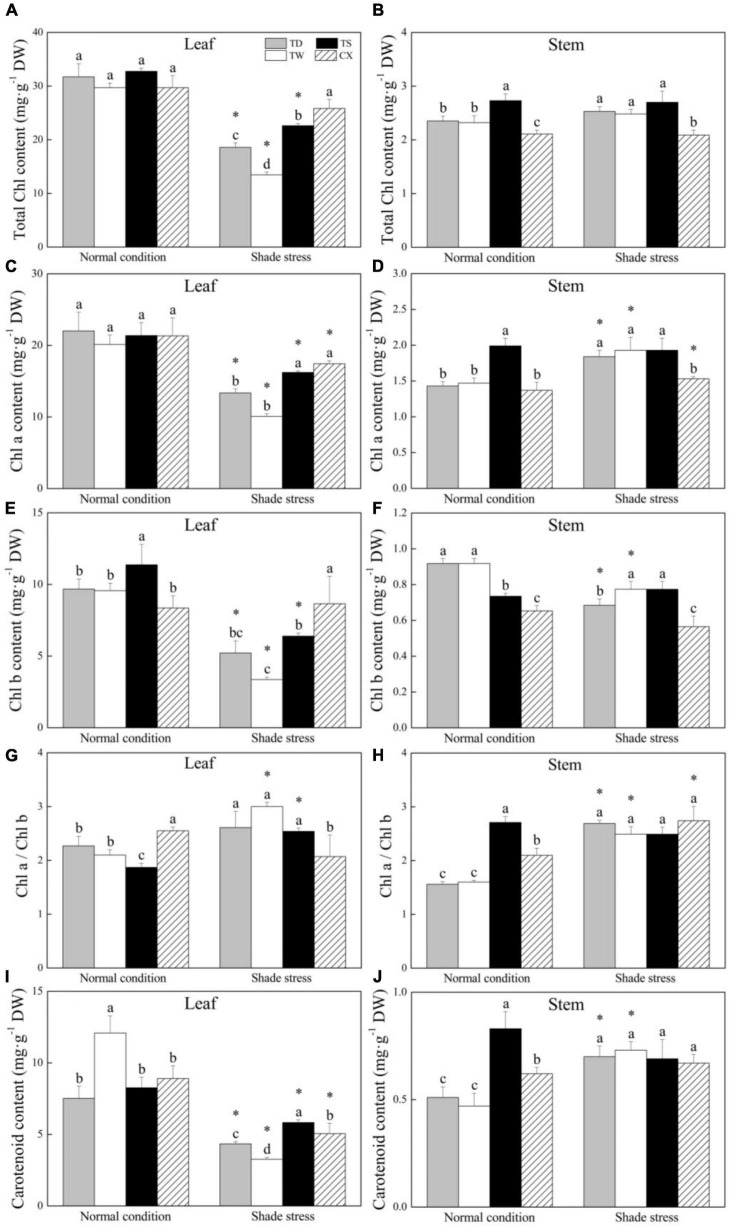
Effects of shade stress on **(A)** total chlorophyll (Chl) a content in leaf, **(B)** total Chl a content in stem, **(C)** Chl a content in leaf, **(D)** Chl a content in stem, **(E)** Chl b content in leaf, **(F)** Chl b in stem, and **(G)** the ratio of Chl a to Chl b in leaf, **(H)** the ratio of Chl a to Chl b in stem, **(I)** carotenoid content in leaf, and **(J)** carotenoid content in stem of four bermudagrass cultivars. Vertical bars indicate the ± SE of mean (*n* = 4). Different letters above columns indicate significant differences (*p* ≤ 0.05) among four cultivars under normal conditions or shade stress. The asterisk (*) above letter indicates a significant difference (*p* ≤ 0.05) in one particular cultivar between normal conditions and shade stress.

Shade stress significantly decreased total Chl in leaves of TD, TW, and TS, but had no significant effect on total Chl content in the CX. The TS and CX maintained significantly higher total Chl content in leaves as compared to the TD and TW under shade stress (Figure 3A). The TS or CX had higher or the lowest total Chl content in stems than TD and TW under normal and shade conditions, respectively (Figure 3B). Shade stress reduced the Chl a content in leaves of common and hybrid cultivars (Figure 3C). The CX and TS had significantly higher Chl a content in leaves than the TD and TW under shade condition (Figure 3C). The Chl a content in stems of the TS was highest under normal conditions, and the TD, TW, and TS exhibited significantly higher Chl a content in stems than the CX under shade stress (Figure 3D). Shade stress significantly decreased Chl b content in leaves of TD, TW, and TS by 46.06, 64.85, and 43.81%, but did not affect the Chl b content in leaves of CX (Figure 3E). For changes in Chl b content in stems, shade stress decreased its content in TD and TW by 25.35 and 15.66%, but did not significantly affect its content in TS and CX (Figure 3F). Under normal conditions, the CX or TS had the highest or the lowest Chl a/b ratio in leaves, respectively (Figure 3G). Under stress conditions, the highest Chl a/b ratio was found in stems of TW (Figure 3G). There were no significant differences in Chl a/b ratio in stems among four cultivars under shade stress (Figure 3H).

The TW had the highest CR content in leaves than the other three cultivars under normal conditions and the CR content significantly declined in all the cultivars after 30 days of shade stress (Figure 3I). The decrement of CR content in leaves was 72.99% in the TW, 42.27% in the TD, 43.12% in the CX, or 29.33% in the TS, respectively (Figure 3I). In stems, the TS maintained significantly higher CR content than other cultivars under normal conditions, and the shade stress led to a significant increase in CR content in the TD and TW, but did not significantly affect CR content in the TS and CX (Figure 3J).

### Changes in Photosynthetic Parameters in Response to Shade

**FIGURE 4:**
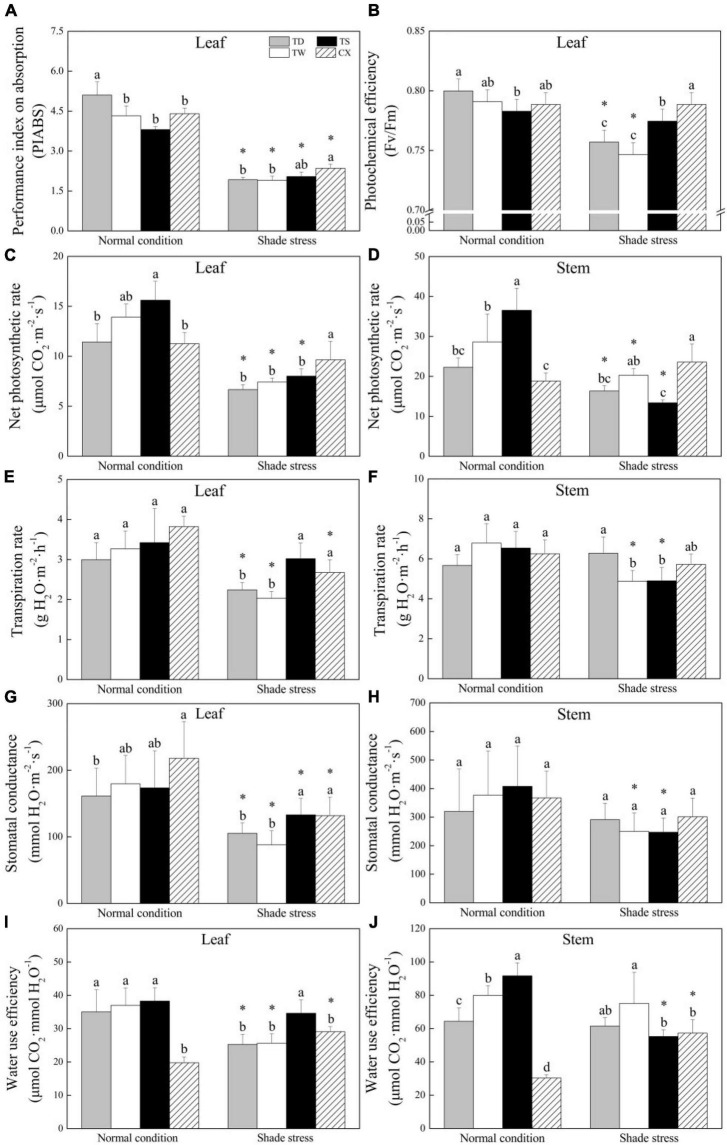
Effects of shade stress on **(A)** performance index on absorption in leaf, **(B)** photochemical efficiency of PS II in leaf, **(C)** net photosynthetic rate in leaf, **(D)** net photosynthetic rate in stem, **(E)** transpiration rate in leaf, **(F)** transpiration rate in stem, **(G)** stomatal conductance in leaf, **(H)** stomatal conductance in stem, and **(I)** water use efficiency in leaf, and **(J)** water use efficiency in stem of four bermudagrass cultivars. Vertical bars indicate the ± SE of mean (*n* = 4). Different letters above columns indicate significant differences (*p* ≤ 0.05) among four cultivars under normal conditions or shade stress. The asterisk (*) above letter indicates a significant difference (*p* ≤ 0.05) in one particular cultivar between normal conditions and shade stress.

The PIABS in leaves of the TD was significantly different from the other three cultivars under normal conditions. Shade stress induced a significant decrease in PIABS of four cultivars, and the CX maintained the highest PIABS than the other cultivars under shade stress (Figure 4A). There was no significant change in Fv/Fm of TS or CX, but the Fv/Fm of TD and TW significantly declined after 30 days of shade stress (Figure 4B). The Pn in leaves and stems of the TD, TW, and TS significantly decreased by 41.60, 46.68, and 48.69% after 30 days of shade stress, but the Pn in leaves of the CX was not significantly changed by the shade stress. The Pn of CX was the highest in leaves and stems under shade stress (Figures 4C,D). Under normal conditions, there were no significant differences in the Tr and Gs in leaves and stems among common and hybrid cultivars (Figures 4E–H). In the leaves, the CX and TS maintained significantly higher Tr and Gs than other cultivars under shade stress (Figures 4E,G). There was no significant difference in Gs among the four cultivars under shade stress in the stems (Figure 4H). The CX had the minimum WUE in both leaves and stems under normal conditions (Figures 4I,J). The TS had significantly higher WUE in leaves as compared with the other three cultivars under shade stress (Figure 4I). The WUE significantly declined by 39.74% or increased by 88.76% in stems of TS or CX after shade stress, respectively (Figure 4J).

### Changes in Organic Metabolites in Response to Shade

**FIGURE 5:**
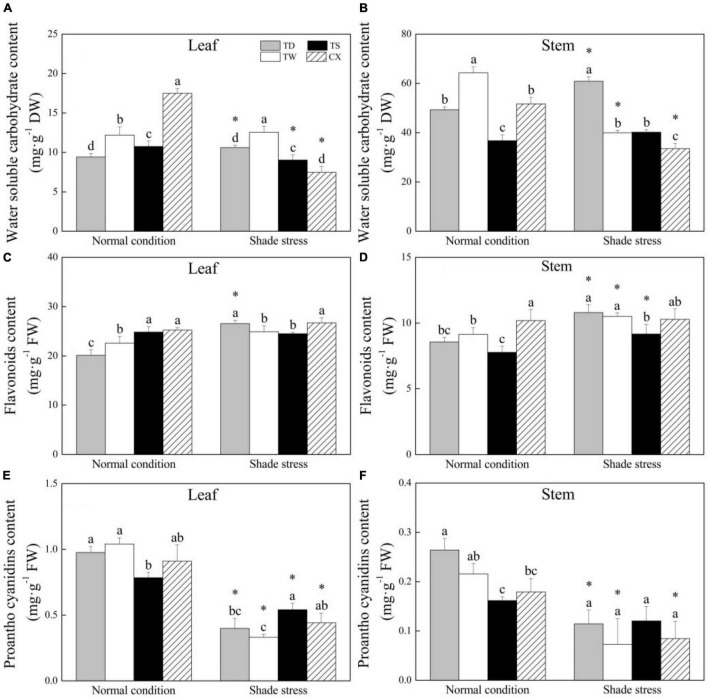
Effects of shade stress on **(A)** water-soluble carbohydrate content in leaf, **(B)** water-soluble carbohydrate content in stem, **(C)** flavonoids content in leaf, **(D)** flavonoids content in stem, and **(E)** proantho cyanidims content in leaf, and **(F)** proantho cyanidims content in stem of four bermudagrass cultivars. Vertical bars indicate the ± SE of mean (*n* = 4). Different letters above columns indicate significant differences (*p* ≤ 0.05) among the four cultivars under normal conditions or shade stress. The asterisk (*) above letter indicates a significant difference (*p* ≤ 0.05) in one particular cultivar between normal conditions and shade stress.

In leaves, the WSC content in CX was the highest and in TD was the lowest among the four cultivars under normal conditions (Figure 5A). Shade stress led to a significant decrease in the WSC content of CX or TS by 57.33 or 15.90%, and a significant increase in TD. The TD and TW exhibited significantly higher WSC in leaves than the TS and CX under shade stress (Figure 5A). In stems, the TW or TS had the highest or lowest WSC content than other cultivars under normal conditions (Figure 5B). Shade stress caused a significant decrease in WSC content in the stems of TW or CX by 37.85 or 35.17%, but a significant increase in TD by 23.4% (Figure 5B). In leaves, the FA content of the TS or the CX was significantly higher than that of the TD and the TW under normal conditions. Under shade stress, the FA content of CX and TD maintained a significantly higher level as compared to the TW and TS (Figure 5C). Shade stress significantly increased the FA content of TD, TW, or TS (Figure 5D). As for PC content, shade stress significantly decreased its content in the leaves of all the cultivars, and the decrement was the biggest in leaves of the TW by 71.11% (Figure 5E). The content of PC in TD, TW, and CX decreased significantly because of shade stress, but there were no significant differences in PC content in the stems of all the cultivars under shade stress (Figure 5F).

### Stress Index, Hierarchical Clustering, and Principal Component Analysis of Four Cultivars in Response to Shade

**FIGURE 6:**
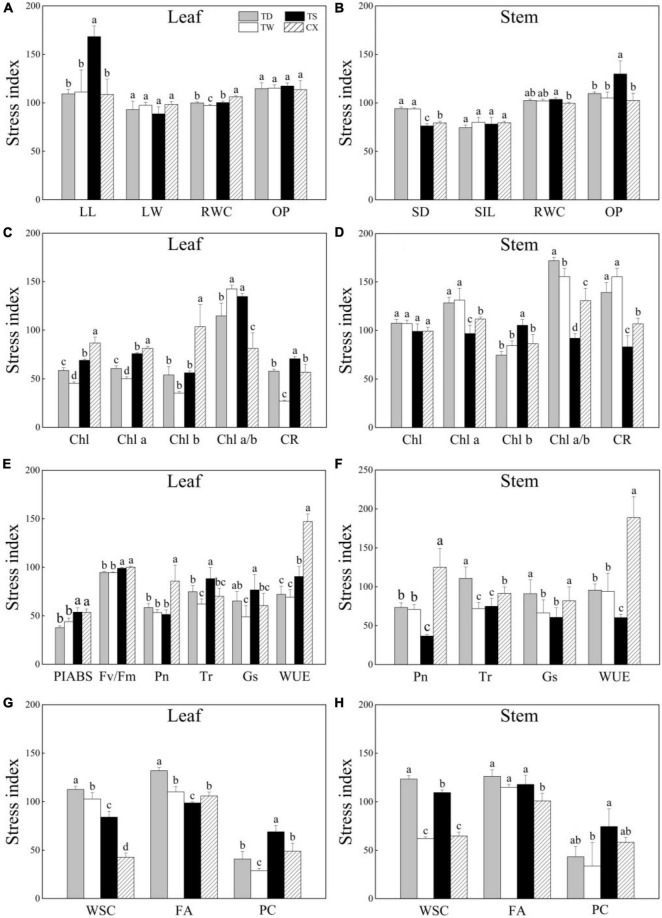
**(A–H)** Effects of shade stress on stress index (SI) of four bermudagrass cultivars. Different letters above columns indicate significant differences (*p* ≤ 0.05). Vertical bars indicate the ± SE of mean (*n* = 4).

The SI of LL was highest in the TS as compared to that in other cultivars and no significant difference was detected in the SI of LW or OP among four cultivars in leaves (Figure 6A). The CX exhibited significantly higher SI of RWC in leaves than other cultivars (Figure 6A). The SI of SD was significantly higher in the TD and TW compared with the SI of TS and CX (Figure 6B). The CX exhibited significantly higher the SI of OP in stems than other cultivars (Figure 6B). The SI of Chl, Chl a, and Chl b in leaves of CX was the highest than the other three cultivars, while the SI of Chl a/b was the lowest (Figure 6C). The TS exhibited the highest or the lowest the SI of Chl b or Chl a/b in stems than other cultivars, respectively (Figure 6D). The SI of Pn and WUE was highest in the leaves of CX as compared to that in other cultivars, and the SI of Tr and Gs was the highest in the leaves of TS (Figure 6E). The CX had the highest SI of Pn and WUE in stems than other cultivars (Figure 6F). The SI of WSC was significantly lower in leaves of CX than that in other cultivars, and the FA of TD was the highest in leaves among four cultivars (Figure 6G). The SI of WSC was significantly lower in the stems of TW and CX than that in the stems of TS and TD, and the SI of FA was the lowest in the stem of CX than other cultivars (Figure 6H). The TS showed the highest SI of PC than other cultivars both in the leaves and stems (Figures 6G,H).

**FIGURE 7:**
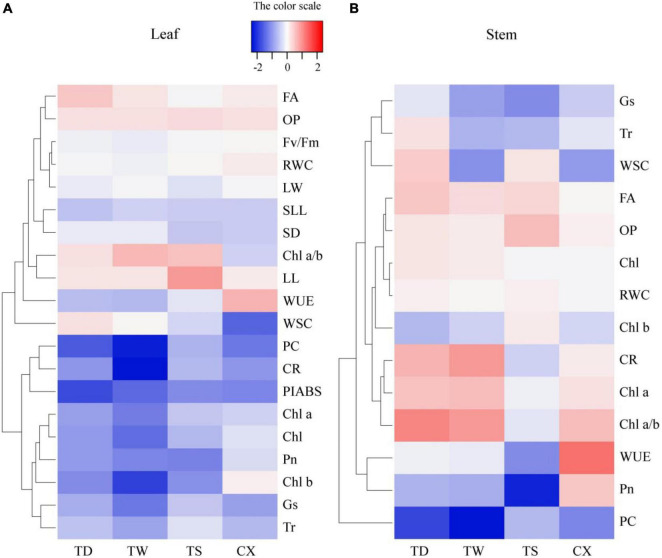
Heatmap and hierarchical clustering for physiological and morphological parameters in **(A)** leaf and **(B)** stem of common and hybrid bermudagrasses.

**FIGURE 8:**
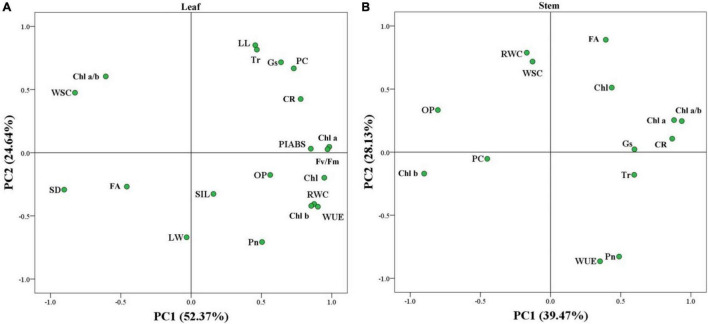
Principal component analysis of the stress index (SI) in **(A)** leaf and **(B)** stem of common and hybrid bermudagrasses.

**FIGURE 9:**
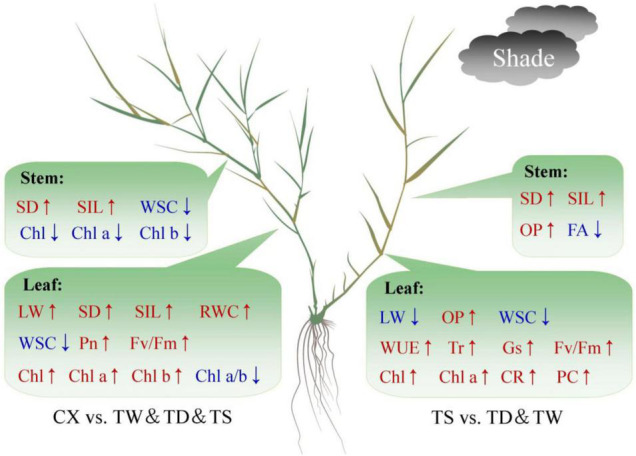
Differential responses to shade stress between common and hybrid bermudagrasses (CX vs. TW&TD&TS) or among three different hybrid bermudagrasses (TS vs. TD&TW).

Hierarchical clustering analysis of 20 parameters in leaves or 14 parameters in stems is shown in the heat map (Figures 7A,B). In leaves, the TS had a more similar variation profile with the CX than TD and TW (Figure 7A). The TD and TW showed similar changes in leaves and stems in response to shade stress (Figures 7A,B). For the PCA, PC1 or PC2 explained 52.37 or 24.64% of the total variance in the leaves, respectively (Figure 8A). OP, SIL, LW, PC, and Pn showed lower variation in leaves than other parameters under shade stress (Figure 8A). In the stems, the PC1 or PC2 explained 39.47 or 28.13% of the total variance, respectively (Figure 8B). Ch a, Chl b, Chl a/b, PC, OP, CC, WUE, and Pn exhibited a bigger variation in stems than other parameters in response to shade stress (Figure 8B). Figure 9 showed differential responses to shade stress between common and hybrid bermudagrasses (CX vs. TW&TD&TS) or among three different hybrid bermudagrasses (TS vs. TD and TW).

## Discussion

Prolonged low-light conditions affect a range of morphological and physiological changes, such as elongated stems and leaves, thinner leaf thickness, decline in OP, and increased tissue water content (Fankhauser and Batschauer, 2016). Accelerated elongation and upward growth as well as increased leaf area were considered to be important adaptive strategies when plants suffer from shade stress, which is known as shadeavoidance syndrome (SAS). These responses provide more possibilities for plants to intercept more light resources for photosynthesis under shade conditions (Carriedo et al., 2016; Fankhauser and Batschauer, 2016). In the current study, shade improved the LL of four bermudagrass cultivars, especially in the TS which showed elongated leaves in response to shade stress. However, the LW of four bermudagrass cultivars was not affected significantly by the shade. Elongated LL could provide more effective areas for light-harvesting and absorption, which was beneficial to maintain higher photosynthesis in the TS under shade stress. Interestingly, the SD and SIL decreased in four bermudagrass cultivars after a prolonged period of shade stress (30 days). It is well known that shade leads to thin and elongated erect stems and tillers in many plant species (Huylenbroeck and Bockstaele, 2001). An earlier study has shown that warm-season grasses are more morphologically restricted by the shade-stress, leading to an overall decrease in turf quality (Xie et al., 2020). Stoloniferous stems of bermudagrasses exhibit creeping habit that improves their aggressiveness for rapid thatch accumulation (Fry and Huang, 2004). The stoloniferous stems are also the main energy storage organ for plantlets regeneration or energy supply and transfer for stress defense (He et al., 2021). This is why stoloniferous stems of four bermudagrass cultivars demonstrated different responses to shade from erect stems in other plant species. In addition, shade stress resulted in a significant decline in OP in leaves of four cultivars, which could be conducive for the maintenance of water balance and turgid cells under stress conditions. Higher RWC and lower OP in stems of the TS and higher RWC in leaves of the CX could be associated with better unfolding of stems or leaves in response to shade stress.

In addition to morphological changes, physiological adaptation (e.g., increased chlorophyll content, decreased ratio of chla/b, or increased ratio of light response center II to light response center I) is also an important strategy for shade-tolerant plants to cope with shade stress (Melis and Harvey, 1981). A short-term relatively lower light intensity can effectively stimulate Chl formation; however, a prolonged period of shade condition inhibits Chl biosynthesis and also accelerates Chl degradation leading to leaf chlorosis (Pires et al., 2011; Huylenbroeck and Bockstaele, 2001). The maintenance of a lower ratio of Chl a/b has been found to be one of the important indicators of stress adaptability in plants (Zeng et al., 2021). Under shade conditions, the proportion of red light decreases and more blue–violet lights are available to the plants. Therefore, the ability to capture and utilize more diffuse light (blue-violet light) will help plants to achieve a dominant position under shade conditions (Dai et al., 2009). As compared to Chl a, the Chl b is capable of absorbing and using more blue-violet lights. Many shade-tolerant plant species decrease the ratio of Ch a/b in response to shade stress. The study of Terashima et al. (2021) showed that the ratio of Chl a/b in leaves of *Alocasia odora* gradually decreased with constantly increasing shade. In our present study, the CX and the TS maintained significantly higher total Chl content and Chl a content than the TD and the TW in leaves. Interestingly, the tetraploid CX exhibited a significantly higher Chl b and a lower ratio of Chl a/b in leaves than triploid three cultivars after 30 days of shade stress. This could be one of the critical adaptive mechanisms in tetraploid bermudagrass as compared to triploid cultivars suffering shade conditions. The previous study has also demonstrated that the Chl a/b ratio in leaves of shade-tolerant warm-season seashore paspalum (*Paspalum vaginatum*) was not affected significantly by low light condition, but it significantly increased in the shade-intolerant TS (Jiang et al., 2005).

Under shade condition, leaf chlorosis and less available long-wavelength lights cause a decline in photosynthesis. Fv/Fm represents the maximum photochemical quantum yield of PSII and is commonly used to illustrate the photochemical efficiency of PSII in plant leaves. PIABS, a key indicator of maximum photochemical efficiency and total numbers of activated photochemical reaction centers of PS II, reflects health status of chloroplasts under stress conditions, such as high-temperature stress, drought, salt stress, and shade stress (Geng et al., 2021; Li et al., 2021; Zeng et al., 2021). Alteration in the PIABS is more sensitive than the Fv/Fm in response to environmental stresses (Yang and Chen, 2015). Although a significant decrease in PIABS was observed in the leaves of all the four bermudagrass cultivars under shade stress, the CX exhibited higher PIABS than the TD, the TW, and the TS. However, shade induced a significant decline in the Fv/Fm in leaves of the TD and the TW, but not in the TS and the CX. A similar result was demonstrated in the study of Jiang et al. (2005) who reported that the Fv/Fm in leaves of TS was not significantly affected after 35 days of low-light stress (60–100 μmol m^–2^ s^–1^).

Photosynthetic capacity, as demonstrated by a change in Pn, is closely related to the maintenance of plant growth and stress tolerance since photosynthesis provides energy supply for plants through CO_2_ assimilation (Pires et al., 2011). In response to environmental stresses, plants adjust stomatal closure and opening to maintain water and gaseous interchange homeostasis (Bharath et al., 2021). Tr often shows a consistent trend with Gs, which is an important indicator of water evaporation and gas exchange (Borel et al., 2010). Our current study found that both Tr and Gs were decreased significantly in four bermudagrass cultivars under shade stress, but the TS and the CX maintained significantly higher Tr and Gs as compared to the TD and the TW, indicating that TS and CX had more frequent gas exchange for photosynthetic maintenance under shade stress. It is noteworthy that a significant decrease in the Pn induced by shade stress was only observed in leaves and stems of triploid hybrid bermudagrass cultivars (TD, TW, and TS). The CX maintained a significantly higher Pn in leaves than TD, TW, and TS under shade stress, which could be related to the maintenance of lower Chl a/b in leaves. In addition, improved WUE in leaves and stems was only observed in the tetraploid CX after 30 days of shade stress. The higher WUE indicates greater energy conversion efficiency associated with dry matter accumulation and water consumption in plants. Zhang et al. (2017) found that the 44% light could improve WUE of walnut (*Juglans regia* L.). The shade-induced significant decrease in WSC accumulation has been found in the bermudagrass and other warm-season grass species (Jiang et al., 2005). Interestingly, the CX and TS did not accumulate more WSC in leaves and stems than the TD and TW under shade stress. The WSC is the main energy donor for maintaining plant growth and development (Liu et al., 2021). These findings suggested that maintenance of higher photosynthesis and WUE were important physiological adaptions to shade stress in the tetraploid CX. The utilization of WSC for maintenance of normal plant growth could be more important than WSC accumulation when bermudagrass species suffered from a prolonged period of shade stress.

Adverse stresses including shade stress induce a large accumulation of ROS, which oxidizes cell membranes and attacks organelles, leading to lipid peroxidation, protein degradation, and impaired function of cytoplasmic organoids (Gill and Tuteja, 2010). CRs are fat-soluble pigments that, to some extent, determine the coloration of organisms. In higher plants, the main role of CRs in photosynthetic organisms is to transmit light energy and quench excess light energy especially under a high light condition (Zakar et al., 2016). CRs also have strong antioxidants that can efficiently kill singlet oxygen and protect the photosynthetic membrane system against oxidative damage (Dewanjee et al., 2021). PC is a member of a large group of FA compounds in plants. Both PC and FA exhibit strong antioxidant capacity and free radical (superoxide anion and hydroxyl radicals) elimination effects for protecting lipids from ROS attack (Fini et al., 2011; Li et al., 2017). A previous study has demonstrated that FA and PC have the ability to improve antioxidant activity and scavenge superoxide anion radicals and hydroxyl radicals to slow down the oxidative damage caused by shade stress (Winkel-Shirley, 2002; Treutter, 2006). As compared to the TD and the TW, the TS and the CX had significantly higher CR content in leaves, which might provide better antioxidant and photoprotection capacities under shade stress. In addition, the maintenance of significantly higher PC content in leaves of the TS could be propitious to adapt to shade environment. However, the tetraploid CX did not have the advantage of the accumulation of FA and PC than triploid hybrid cultivars in response to shade stress.

## Conclusion

In conclusion, common (CX) and hybrid (TW, TS, and CX) bermudagrasses exhibited some common and differential responses to shade stress. In leaves, the TS had a more similar variation profile with CX than TD and TW, whereas TD and TW showed similar changes in leaves and stems under shade stress. Among the three hybrids (TW, TS, and CX) bermudagrasses, an increase in LL and maintenance of higher photosynthetic performance (higher Fv/Fm, total Chl content, and Chl a content), gas exchange (higher Tr and Gs), and WUE in leaves could be better morphological and physiological adaptations of TS to shade than other hybrid bermudagrass (TD and TW). The TS also had significantly higher CR and PC contents than the TD and the TW, which indicated a potentially better antioxidant capacity in leaves of the TS under shade stress (Figure 9). In addition, tetraploid common CX showed excellent shade tolerance than triploid hybrid TD, TW, and TS mainly by maintaining higher effective photosynthetic leaf area (higher LW), photosynthetic performance of PSI and PSII (higher Pn and Fv/Fm), and photosynthetic pigments and ratio (lower Chl a/b ratio, higher total Chl, Chl a, and Chl b content) for efficient use of light energy under shade stress (Figure 9). Furthermore, OP, SIL, LW, PC, and Pn showed lower variation in leaves than other parameters, and Ch a, Chl b, Chl a/b, PC, OP, CC, WUE, and Pn exhibited bigger variation in stems than other parameters under shade stress. Leaves showed more variations than stems among the four bermudagrasses in response to shade stress, which indicated the physiological importance of leaves when warm-season bermudagrasses suffered from the shade.

## Data Availability Statement

The raw data supporting the conclusions of this article will be made available by the authors, without undue reservation.

## Author Contributions

ZL conceived and designed the research. YC and KY conducted the experiments. ZL and YC evaluated the data and completed the manuscript writing. YP provided different chemical reagents and experimental material. GF, WL, and YP reviewed and edited the manuscript. All authors contributed to the article and approved the submitted version.

## Conflict of Interest

The authors declare that the research was conducted in the absence of any commercial or financial relationships that could be construed as a potential conflict of interest.

## Publisher’s Note

All claims expressed in this article are solely those of the authors and do not necessarily represent those of their affiliated organizations, or those of the publisher, the editors and the reviewers. Any product that may be evaluated in this article, or claim that may be made by its manufacturer, is not guaranteed or endorsed by the publisher.
